# *Candida* Bloodstream Infection: Changing Pattern of Occurrence and Antifungal Susceptibility over 10 Years in a Tertiary Care Saudi Hospital

**DOI:** 10.1155/2019/2015692

**Published:** 2019-12-17

**Authors:** Nawaf Alkharashi, Sameera Aljohani, Laila Layqah, Emad Masuadi, Waleed Baharoon, Hamdan AL-Jahdali, Salim Baharoon

**Affiliations:** ^1^Department of Emergency Medicine, King Saud Bin Abdulaziz University for Health Sciences, Riyadh, Saudi Arabia; ^2^Department of Pathology and Laboratory Medicine, King Abdulaziz Medical City, Riyadh, Saudi Arabia; ^3^Department of Research Office, King Abdullah International Medical Research Center, Riyadh, Saudi Arabia; ^4^College of Medicine, King Saud Bin Abdulaziz University for Health Sciences, Riyadh, Saudi Arabia; ^5^College of Dentistry, King Saud Bin Abdulaziz University for Health Sciences, Riyadh, Saudi Arabia; ^6^Department of Medicine, King Abdulaziz Medical City, Riyadh, Saudi Arabia; ^7^Department of Intensive Care, King Abdulaziz Medical City, Riyadh, Saudi Arabia

## Abstract

**Background:**

*Candida* has emerged as one of the most important pathogens that cause bloodstream infection (BSI).Understanding the current *Candida* BSI trends, the dominant species causing disease and the mortality associated with this infection are crucial to optimize therapeutic and prophylaxis measures.

**Objectives:**

To study the epidemiology and to evaluate the risk factors, prognostic factors, and mortality associated with candidemia and to compare these findings with previously published studies from Saudi Arabia.

**Design:**

A retrospective medical record review.

**Setting:**

Tertiary hospital in Riyadh.

**Patients and Methods:**

The analysis included all cases of *Candida* blood stream infection who are >18 years old over the period from 2013 to 2018. Continuous variables were compared using the parametric *T*-test while categorical variables were compared using the Chi-squared test.

**Main Outcome Measure:**

Incidence, resistance, and hospital outcomes in *Candida* blood stream infection.

**Sample Size:**

324 patients.

**Results:**

Three hundred and twenty-four episodes of *Candida* blood stream infections were identified. Median age of patients was 49.7 SD ± 28.1 years, and 53% of patients were males. More than half of the patients had an underlying disease involving the abdomen or laparotomy, 78% had an indwelling intravenous catheter, and 62% had suffered a bacterial infection within 2 weeks prior to candidemia. *Candida albicans* represents 33% of all isolates with decreasing trend overtime. There was an increase in the number of non*albicans Candida* overtime with *Candida tropicalis* in the lead (20%). Use of broad spectrum antibiotics (82%), prior ICU admission (60%) and use of central venous catheters (58%) were the most prevalent predisposing factors of candidemia. Azole resistance was variable overtime. Resistance to caspofungin remained very low (1.9%). Fourteen days crude mortality was 37% for ICU patients and 26.7% in non-ICU patients, while hospital crude mortality was 64.4% and 46.7%, respectively.

**Conclusion:**

There is an increasing trend of non*albicans Candida* blood stream infection. Fluconazole resistance remained low to *C. albicans*. Most isolates remain susceptible to caspofungin, voriconazole, and amphotericin B. *Candida* bloodstream infection is associated with high 14-day hospital mortality.

## 1. Introduction

Over the last two decades, *Candida* has emerged as one of the most important pathogens causing nosocomial bloodstream infection in both adults and children worldwide [1–6]. *Candida* is part of our normal flora, and more than 200 species have been described, but only 10% are known to cause human infections [7]. In hospitalized patients and especially in the critically ill patients, *Candida* is between the fourth and sixth most common isolated pathogen in bloodstream infections [8–12].

As a single species, *C. albicans* accounts for close to 50% of overall invasive *Candida* infection. However, there has been a proportionate increase in the isolation of non*albicans* species of *Candida* [4, 13–18].

Incidence of *Candida*-invasive blood infection and *Candida* species isolated varies according to patient population and geographical locations. While some surveillance has described an increase in the incidence of candidemia, others have showed either a stable or decreasing trends [19–24].

In Saudi Arabia, candidemia incidence is not precisely known. Earlier studies revealed a low incidence in general ranging between 0.2 and 0.76 cases/1000 hospital discharges, [25–28] while more recent studies revealed a higher incidence with a median rate of 1.65 per 1,000 hospital discharges per year with a significant trend towards higher rates over time [29, 30]. *Candida* accounts for 2.8% of all positive blood cultures [31].

The reported mortality secondary to candidemia ranges from 30 to 60% with up to 30 days increase in the length of hospital stay for survivors [11, 12, 16, 32, 33].

Risk factors of bloodstream infections with *Candida* species have been extensively studied and include malignancies, neutropenia, prolonged ICU (intensive care unit) stays, *Candida* colonization, severe illness, diabetes, renal failure, hemodialysis, receipt of prolonged courses of broad-spectrum antibiotics, central venous catheterization, parenteral hyperalimentation, immunosuppressive drugs, and transplantation [34–38].

The current project aims to study the epidemiology and to evaluate the risk factors, prognostic factors, and mortality associated with candidemia and to compare these findings with previously published studies from Saudi Arabia.

## 2. Method

This is a retrospective analysis of all cases of *Candida* blood stream infection over the period from 2008 to 2015 from a tertiary care hospital in Riyadh Saudi Arabia. National Guard (NGHA) hospital in Riyadh is multiple specialty hospital with a total bed capacity of more than 1200 beds.

*Candida* blood stream infection is defined as at least 1 blood culture positive for *Candida* species for a patient who developed signs and symptoms of BSI >48 h after hospital admission. Only the first episode of candidemia was included.

Demographic and clinical data of age, gender, primary illness, comorbidities, and risk factors such as duration of antibiotic therapy, intravenous catheters, endotracheal intubation, and mechanical ventilation at the time when blood culture was positive were all collected.

When data were available, we calculated the *Candida* score for patients. The score consists of the following: multifocal *Candida* colonization (1 point), surgery on ICU admission (1 point), severe sepsis (2 points), and TPN (1 point). A cutoff of more than or equal to three was highly predictive of fungal infection. The score is created based on the four predictors of invasive fungal infection in the Estudio de Prevalencia de CANdidiasis project [39]. There was a significant linear association between higher values and invasive fungal infection especially in ICU patients, and a higher score could be used to risk stratify patients for early antifungal treatment [40]. *Candida* colonization data were frequently missing especially in non-ICU patients.

*Candida* identification was carried out via VITEK® 2 (bioMérieux, Inc. Hazelwood, MO, USA) healthcare system and bioMérieux API 20C AUX, a system for the identification of the most frequently encountered yeasts. *Candida* susceptibility was primarily performed with bioMérieux VITEK® 2 Fungal Susceptibility (AST-Y07). Thermo Scientific™ Sensititre™ YeastOne™ YO10 AST antifungal testing (colorimetric microplate-based assay) was occasionally used. Both methods have shown good agreement with the Clinical and Laboratory Standards Institute (CLSI) broth microdilution reference method (BMD) [39–46].

The permission of the Ethics Committee at King Abdullah International Medical Research Center (KAIMRC) was obtained.

## 3. Statistical Analysis

Standard descriptive statistics were used. Categorical data were reported as frequencies and percentages, while continuous variables were reported as mean ± standard deviation. Continuous variables were compared using the parametric *T*-test while categorical variables were compared using the Chi-squared test. Multivariate logistic regression was used to the assess C*andida* risk factors. Tests were performed two-tailed and considered significant when *p* value  <0.05. All statistical tests were performed using the statistical package IBM SPSS for Windows (version 20.0: SPSS, Chicago, IL, USA).

## 4. Results

Over the study period, a total of 324 patients with candidemia were identified. Male-to-female ratio was 1.14 with a mean age of 49.7 SD ± 28.1. *Candida albicans* was the leading cause of candidemia across all years accounting for 33%. Non*albicans* strains as a group were more common representing 67% of all isolates (Table 1).

More than two thirds of candidemia episodes (67.6%) occurred in the intensive care units (ICUs) followed by medical wards (15%). There were more candidemia episodes from cardiac wards (6.5%) including CCU and medical cardiac ICU compared with surgical (5.6%) and hematology (5.2%) wards.

**Figure 1:**
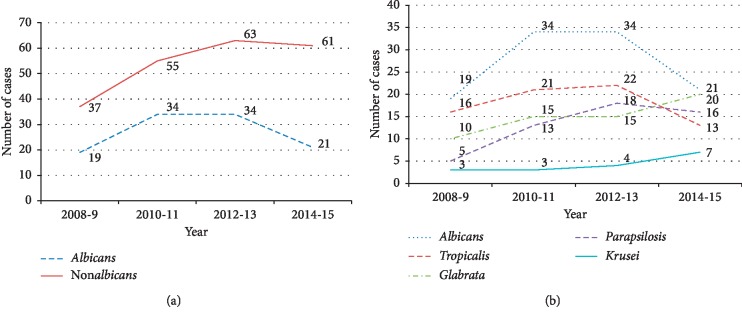
Trends of candidemia over time. (a) *Albicans* vs non*albicans. *(b) *Candida* spp.

**Figure 2:**
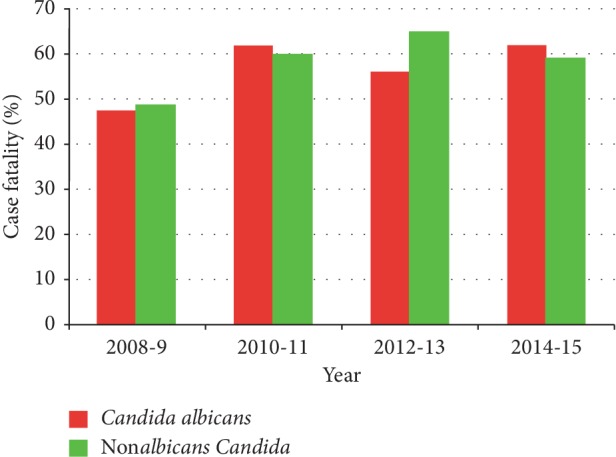
Hospital case fatality.

In the first two years of the study, there was an increase in candidemia of both non*albicans* and *C*. *albicans* groups. While the rate of candidemia due to *C. albicans* was stable between 2010 and 2013 and decreasing thereafter, non*albicans* candidemia continues to increase (Figure 1(a)). *Candida tropicalis* followed by *Candida glabrata* and *Candida parapsilosis* were the most commonly isolated in the non*albicans* group. While number of isolates due to *C. tropicalis* was decreasing, both *C. glabrata* and *C. krusei* were on the rise (Figure 1(b)). Non*albicans* group were more frequently isolated in ICU patients (63.5% vs. 37.3%, *p* 0.078) crude mortality within the first two weeks after candidemia was 64% and is more observed among patients in ICU when the diagnosis is made (37% vs. 27% *p* 0.016) (Table 2). Overall hospital mortality was 59%. Crude mortality remained high for both non*albicans* and *C*. *albicans* groups with a slightly lower rate for former overtime (Figure 2).

Patients where candidemia was diagnosed in ICU were significantly less likely to leave hospital alive (*p* 0.002) (Table 2) Older age, candidemia in the patients with chronic liver disease, and treatment with azole therapy were all associated with worst outcome, while invasive *Candida* infection in trauma/surgery patients and those that are device-related have a better outcome (Table 2).

In multivariate analysis, risk factors for candidemia includes use of broad-spectrum antibiotics (81.5%) followed by ICU admission (60.2%) and use of central venous catheters (58%) (Table 3). *Candida* score was less or equal to 2 in 79% of patient with candidemia.

**Figure 3:**
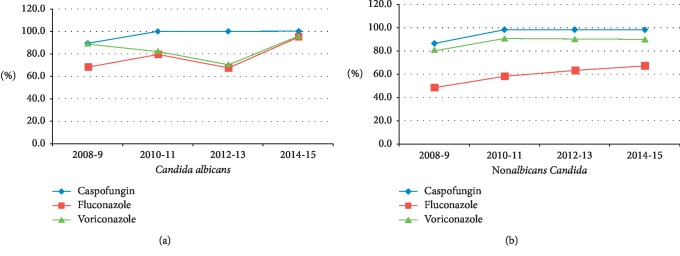
Susceptibility trend over time.

The *Candida albicans* group remained very susceptible to amphotericin B and echinocandin (caspofungin was the only echinocandin available in our hospital during the study period) (Table 4). Susceptibility to fluconazole remained high (77%). Among non*albicans* group susceptibility to fluconazole and voriconazole were 60% and 89%, respectively (Table 4). Although susceptibility to azoles (fluconazole and voriconazole) among the *C*. *albicans* group was trending lower during the study period, there was a significant increase in susceptibility over time in recent years in both *C. albicans* and non*albicans* groups (Figures 3(a) and 3(b)).

## 5. Discussion

*Candida* infection is a leading cause of invasive fungal infection worldwide [1, 2, 4, 13, 30]. Epidemiological studies have suggested that the annual incidence of candidemia in some countries might have stabilized or even decreased; however, there is a significant geographical variation [2, 4, 14, 18, 22–25, 29, 30].

Local epidemiological surveillance studies are important to guide empirical and therapeutic antifungal therapy. There is no Saudi national data on incidence and prevalence of invasive fungal infection. However, some centers have reported low and decreasing trends, while others showed an increasing rate [22–25, 30]. *Candida albicans*-invasive infection remains the most frequently isolated single species in our study albeit trending down frequency. Similar to other studies, BSI due to non*albicans Candida* as a group is higher with increasing frequency [29, 47, 48]. *Candida tropicalis* is the most frequently isolated among the non*albicans* group. In Saudi Arabia, *Candida tropicalis* has been the main species isolated among NAC (non*albicans Candida*) in both adult and pediatric population in most of the studies reported followed by *Candida glabrata* [6, 25–27, 30]. Risk factors for the emergence of non*albicans Candida* include increasing use of an antifungal regimen specially fluconazole, use of broad-spectrum antibiotics, and the increasing number of immunocompromised patients [37, 49, 50]. The decreasing trends of *Candida tropicalis* over time in our cohort is substituted by increasing frequency of *C. glabrata* and *C. Krusei.* This change over time may reflect patient variation and antimicrobial regimens that include more echinocandin use [51].

The European SENTRY investigators' reported *C. parapsilosis* as the most frequently encountered *Candida* spp, while *C. glabrata* as the most commonly isolated NAC in US [2]. Other *Candida* species were more predominant in other countries. Such variability likely represents differences in populations studied and risk factors encountered [4, 32, 52].

Risk factors for invasive *Candida* across many studies from Saudi are consistent and similar to what is reported internationally. Use of broad-spectrum antibiotics, admission to ICU, and central vascular access were the main reported [6, 29, 30, 53].

Extensive use of broad-spectrum antimicrobial remains a very big challenge in Saudi Arabia. Ministry of health has recently launched a major campaign to combat the crisis of inappropriate use of antimicrobial in the Kingdom. More than two-third of our patients were ICU patients or with previous visit to ICU which is a major place for antimicrobial use. Vascular devices were in place in 58% of patients with candidemia. Those two factors are amenable to improvement through effective stewardship programs.

Most of the *Candida* spp. remains sensitive to polyene and echinocandins worldwide [11, 30, 54]. *Candida albicans* remains mostly sensitive to azoles. Resistance to fluconazole ranges between 0.3 and 2 percent [2, 53, 54]. However, *Candida albicans* with reduced susceptibility to fluconazole have been observed in many centers including Saudi Arabia [31, 55]. In our series, only 68% of *Candida albicans* isolates were reported sensitive to fluconazole at the start of the study, but much higher susceptibility was observed at the end of the study (95%). Similar to other studies, resistance to fluconazole was overall predictive of resistance to voriconazole in our series [54, 56]. *Candida Krusei* susceptibility to amphotericin B was lower than what is reported internationally but consistent with what was previously reported from Saudi Arabia (76%) [11, 29, 56].

Invasive *Candida* infection is associated with significant mortality especially in ICU and among older patients [1, 4, 11, 16, 30, 32, 33, 57, 58]. Both hospital and 14-day mortality in our cohort was high and was significantly higher among patients with ICU candidemia (37% vs. 26% *p* 0.066) and in those with candidemia related to vascular device. Patients with chronic liver disease and chronic and or acute renal failure requiring renal supportive therapy have significantly worse outcomes (*p* 0.017 and 0.003) Treatment for less than 48 hours and with azole therapy were also associated with worse outcome.

This study still represents single center experience which may vary according to hospital profile of admission and regional patient's characteristics. There is a need for more comprehensive national data that should not be limited to one health care provider or geographical areas.

In conclusion, the non*albicans Candida* group was the major cause of invasive candidemia and was trending higher overtime while *Candida albicans* were decreasing. *Candida glabrata* is emerging as the most frequent overtime. Most of the *Candida* spp. remained highly susceptible to all lines of therapy. Mortality remained high for all cases with invasive candidemia and especially among critically ill patients.

## Figures and Tables

**Table 1:** Patients general characteristics.

Item	Identified variables	*N* (%)
Gender	Male	173 (53.4)
	Female	151 (46.6)
Age	Mean ± SD	49.7 ± 28.1
Place of isolation	Intensive care unit (ICU)	219 (67.6)
	Medical	49 (15)
	Others^*∗*^	56 (17.3)
	Nonintensive care unit	105 (32.4)
Risk factors	Prior ICU admission	195 (60.2)
	Neutropenia	19 (5.9)
	Use of broad-spectrum antibiotic	264 (81.5)
	Presence of vascular device	188 (58)
	Internal jugular	98 (30.2)
	Subclavian	34 (10.5)
	Peripherally inserted central catheter (PICC)	33 (10.2)
	Femoral	64 (19.8)
	Parenteral nutrition	60 (18.5)
	Intra-abdominal infection	15 (4.6)
	Others (medications)	44 (13.6)
*Candida* species	*C. albicans*	108 (33.3)
	Non*albicans*	216 (66.7)
	*C. tropicalis*	72 (22.2)
	*C. glabrata*	60 (18.5)
	*C. parapsilosis*	52 (16)
	*C. krusei*	17 (5.2)
	Others	15 (4.6)
Drug susceptibility profile (susceptible)	Amphotericin B	315 (97.2)
	Caspofungin	314 (96.9)
	Fluconazole	214 (66)
	Voriconazole	281 (86.7)
14 days outcome	Alive	215 (66.4)
	Dead	109 (33.6)
Hospital outcome	Alive	134 (41.4)
	Dead	190 (58.6)

^*∗*^= surgical 5.6%, cardiac 6.5%, and hematology 5.2%.

**Table 2:** Patients outcome.

Identified variables	Variable	14 days postisolation outcome			Hospital outcome		
		Dead *N* (%)	Alive *N* (%)	*p* value	Dead *N* (%)	Alive *N* (%)	*p* value
Age	≤18	15 (23.4)	49 (76.6)	0.054	26 (40.6)	38 (59.4)	0.001
	>18	94 (36.2)	166 (63.8)		164 (63.1)	96 (36.9)	
Mean age ± SD		54.9 ± 26	47 ± 28.82	0.013	55.7 ± 26.2	41.2 ± 28.7	<0.001
Gender	Male	60 (34.7)	113 (65.5)	0.671	102 (59)	71 (41)	0.901
	Female	49 (32.5)	102 (67.5)		88 (58.3)	63 (41.7)	
Primary diagnosis	Abdominal pathology	13 (28.3)	33 (71.7)	0.404	29 (63)	17 (37)	0.513
		96 (34.5)	182 (65.5)		161 (57.9)	117 (42.1)	
	Malignancy	23 (37.1)	39 (62.9)	0.522	39 (62.9)	23 (37.1)	0.449
		86 (32.8)	176 (67.2)		151 (57.9)	111 (42.4)	
	Trauma/surgery	4 (13.8)	25 (86.2)	0.018	11 (37.9)	18 (62.1)	0.018
		105 (35.6)	190 (64.4)		179 (60.7)	116 (39.3)	
	Sepsis/infection	40 (40.8)	58 (59.2)	0.072	60 (61.2)	38 (38.8)	0.534
		69 (30.5)	157 (69.5)		130 (57.5)	96 (42.5)	
	Kidney disease	12 (32.4)	25 (67.6)	0.869	22 (59.5)	15 (40.5)	0.915
		97(33.8)	190 (66.2)		168 (58.5)	119 (41.5)	
	Burn	4 (30.8)	9 (69.2)	0.823	7 (53.8)	6 (46.2)	0.720
		105 (33.8)	206 (66.2)		183 (58.8)	128 (41.2)	
	Others	36 (32.1)	76 (67.9)	0.678	67 (59.8)	45 (40.2)	0.754
		73 (34.4)	139 (65.6)		123 (58)	89 (42)	
Comorbidities	Diabetes mellitus	59 (35.8)	106 (64.2)	0.412	113 (68.5)	52 (31.5)	<0.001
		50 (31.4)	109 (68.6)		77 (48.4)	82 (51.6)	
	Renal disease	48 (44.9)	59 (55.1)	0.003	79 (73.8)	28 (26.2)	<0.001
		61 (28.1)	156 (71.9)		111 (51.2)	106 (48.8)	
	Cardiac disease	24 (32)	51 (68.1)	0.731	55 (73.3)	20 (26.7)	0.003
		85 (34.1)	164 (65.9)		135 (54.2)	114 (45.8)	
	Respiratory disease	16 (37.2)	27 (62.8)	0.595	27 (62.8)	16 (37.2)	0.553
		93 (33.1)	188 (66.9)		163 (58)	118 (42)	
	Liver disease	16 (53.3)	14 (46.7)	0.017	24 (80)	6 (20)	0.013
		93 (31.6)	201 (68.4)		166 (56.5)	128 (43.5)	
	Malignancy	20 (35.1)	37 (64.9)	0.799	32 (56.1)	25 (43.9)	0.673
		89 (33.3)	178 (66.7)		158 (59.2)	109 (40.8)	
	Recent steroid use	62 (39.2)	96 (60.8)	0.037	106 (67.1)	52 (32.9)	0.003
		47 (28.3)	119 (71.7)		84 (50.6)	82 (49.4)	
	Others	5 (33.3)	10 (66.7)	0.979	179 (57.9)	4 (26.7)	0.237
		104 (33.7)	205 (66.3		11 (73.3)	130 (42.1)	
Site at isolation	ICU	81 (37)	138 (63)	0.066	141 (64.4)	78 (35.6)	0.002
	Non-ICU	28 (26.7)	77 (73.3)		49 (46.7)	56 (53.3)	
Device related	Yes	76 (40.4)	112 (59.6)	0.002	124 (66)	64 (43)	0.002
	No	33 (24.3)	103 (75.7)		66 (48.5)	70 (51.5)	
Prior ICU admission	Yes	68 (34.9)	127 (65.1)	0.565	121 (62.1)	74 (37.9)	0.126
	No	41 (31.8)	88 (68.2)		69 (53.5)	60 (46.5)	
Use of broad-spectrum antibiotics	Yes	95 (36)	169 (64)	0.061	168 (88.4)	96 (71.6)	<0.001
	No	14 (23.3)	46 (76.7)				
*Candida* species	*C. albicans*	40 (37)	68 (63)	0.322	46 (42.6)	62 (57.4)	0.666
	Non*albicans*						
	(i) *C. tropicalis*	69 (31.9)	147 (68)		88 (40.7)	128 (59.3)	
	(ii) *C. glabrata*	21 (29.2)	51 (70.8)		42 (58.3)	30 (41.7)	
	(iii) *C. parapsilosis*	19 (31.7)	41 (68.3)		40 (66.7)	20 (33.3)	
	(iv) *C. krusei*	15 (28.8)	37 (71.2)		28 (53.8)	24 (46.2)	
		9 (52.9)	8 (47.1)		11 (64.7)	6 (35.3)	
Risk factors	Yes	105 (33.5)	208 (66.5)	0.846	186 (58.4)	127 (40.6)	0.127
	No	4 (36.4)	7 (63.6)		4 (36.4)	7 (63.6)	
Prior colonization	Yes	35 (35)	65 (65)	0.730	62 (62)	38 (38)	0.412
	No	74 (33)	150 (67)		128 (57.1)	96 (42.9)	
Treatment duration	≤48 h	18 (69.2)	8 (30.8)	0.001	21 (80.8)	5 (19.2)	0.017
	>48 h	83 (29.5)	198 (70.5)		159 (56.6)	122 (43.4)	
Azole therapy	Yes	15 (20)	60 (80)	0.004	33 (44)	42 (56)	0.003
	No	94 (37.8)	155 (62.2)		157 (63.1)	92 (36.9)	

**Table 3:** A multivariate regression analysis of *Candida* risk factors.

Variable	Infection outcome				Hospital outcome			
	95% CI for OR				95% CI for OR			
	Lower	Upper	*p* value	OR	Lower	Upper	*p* value	OR
Prior ICU admission (yes/no)	0.531	1.478	0.642	0.886	0.71	1.89	0.559	1.16
Neutropenia (yes/no)	0.218	1.936	0.439	0.65	0.29	2.01	0.585	0.76
Use of broad-spectrum antibiotic (yes/no)	0.948	3.609	0.071	1.849	1.74	5.77	<0.001	3.17
CV (yes/no)	1.269	3.631	0.004	2.146	1.22	3.26	0.006	1.99
TPN (yes/no)	0.286	1.085	0.085	0.557	0.56	1.86	0.944	1.02
Chemotherapy (yes/no)	0.267	2.683	0.778	0.847	0.33	2.61	0.887	0.93
Intra-abdominal infection (yes/no)	0.426	3.865	0.658	1.283	0.37	3.43	0.834	1.13
Chronic use of steroid (yes/no)	0.36	4.03	0.762	1.205	0.16	1.68	0.267	0.51
Immune-modulating drugs (yes/no)	0.285	5.194	0.792	1.216	0.16	2.55	0.527	0.64

**Table 4:** *Candida* species susceptibility profile.

*Candida* spp	Amphotericin B N (%)	Caspofungin N (%)	Fluconazole N (%)	Voriconazole N (%)
*C. albicans*	106 (98.1)	106 (98.1)	83 (76.9)	89 (82.4)
*C. tropicalis*	72 (100)	69 (95.8)	52 (72.2)	63 (87.5)
*C. glabrata*	60 (100)	58 (96.7)	29 (48.3)	47 (78.3)
*C. parapsilosis*	52 (100)	52 (100)	32 (61.5)	51 (98)
*C. krusei*	13 (76.5)	16 (94)	4 (23.5)	16 (94)
Others	12 (80)	13 (86.7)	14 (93.3)	15 (100)

## Data Availability

The data used to support the findings of this study are available from the corresponding author upon request.

## Additional Points

This study still represents single-center experience which may vary according to the hospital profile of admission and regional patient's characteristics.
